# Erfassung der Mundgesundheit von ambulant betreuten Senioren durch Hausärzte: Entwicklung und Validierung des geriatrischen ambulanten Mundgesundheits-Screenings

**DOI:** 10.1007/s00391-020-01730-5

**Published:** 2020-04-29

**Authors:** Anna Greta Barbe, Sabine Spiritus, Anna Hagemeier, Michael J. Noack, Gabriele Röhrig

**Affiliations:** 1 Fachbereich Parodontologie, AG Seniorenzahnmedizin, Poliklinik für Zahnerhaltung u. Parodontologie, Uniklinik Köln, Kerpener Str. 32, 50931 Köln, Deutschland; 2 Institute of Medical Statistics and Computational Biology, Medical Faculty, University of Cologne, 50924 Köln, Deutschland; 3 Zentrum für spezialisierte geriatrische Diagnostik, MVZ Medicum Köln Ost, Johann-Classen-Straße 68, 51103 Köln, Deutschland

**Keywords:** Alterszahnmedizin, Kaufunktion, Mangelernährung, Geriatrische Syndrome, Mundtrockenheit, Geriatric dentistry, Chewing function, Malnutrition, Geriatric syndromes, Xerostomia

## Abstract

**Hintergrund:**

Einschränkungen der Mundgesundheit ambulant betreuter Senioren in Deutschland werden trotz hoher Prävalenz im hausärztlich-geriatrischen Bereich nicht routinemäßig erfasst. Da Senioren Hausärzte mit höherem Alter häufiger aufsuchen als Zahnärzte, stellt sich die Frage, ob reduzierte Mundgesundheit im Praxisalltag mit einem interdisziplinären Screeninginstrument identifiziert werden kann.

**Ziel der Arbeit:**

Ziel der Arbeit ist, ein Screeninginstrument für reduzierte Mundgesundheit für Hausärzte zu entwickeln und dieses durch zahnmedizinische Befunde zu validieren.

**Material und Methoden:**

Das geriatrische ambulante Mundgesundheits-Screening (GAMS) als subjektives Screeninginstrument wurde entwickelt, um für geriatrische Patienten relevante zahnmedizinische Aspekte wie Kauprobleme, Schmerzen, Parodontitis, Mundgeruch oder Mundtrockenheit in dichotomen Fragen abzubilden. Zudem erfolgt die Einschätzung der Dringlichkeit eines Zahnarztbesuches durch den Behandler. Es wurden *n* = 75 Patienten eingeschlossen und der GAMS sowie eine zahnärztliche Untersuchung zur Validierung durchgeführt.

**Ergebnisse:**

Bei subjektiver Einschätzung als auch im zahnmedizinischen Befund zeigte sich reduzierte Mundgesundheit, insbesondere bei Risikofaktoren für die Entwicklung systemischer Komorbiditäten wie Dysphagie und Mangelernährung, wobei Mundgesundheitsprobleme durch die Patienten unterschätzt wurden. Einbisse, Kauschwierigkeiten und Mundtrockenheit zeigten ausreichende Übereinstimmung zwischen oralem Befund und subjektiver Einschätzung.

**Diskussion:**

Der GAMS könnte beitragen, die Erwägung und Berücksichtigung von Mundgesundheitsproblemen bei geriatrischen Patienten im hausärztlichen Setting zu erleichtern und die Zusammenarbeit mit Zahnmedizinern im Sinne europäischer Handlungsempfehlungen fördern.

**Zusatzmaterial online:**

Zusätzliche Informationen sind in der Online-Version dieses Artikels (10.1007/s00391-020-01730-5) enthalten.

## Abstract (en)

**Background:**

Despite a high prevalence in the general practitioner (GP) and geriatric field, limitations of oral health of seniors under outpatient care in Germany are not routinely recorded. Since seniors with a high age visit GPs more often than dentists, the question arises whether an interdisciplinary screening instrument can be used to identify reduced oral health in routine practice.

**Objective:**

The aim of the work was to develop a screening tool for reduced oral health for GPs and to validate this by dental examinations.

**Material and methods:**

The geriatric outpatient oral health screening (GAMS) was developed as a subjective screening instrument to depict dental aspects relevant for geriatric patients, such as chewing problems, pain, periodontitis, bad breath or dry mouth in dichotomous questions. The urgency of a visit to the dentist is also assessed by the family doctor. A total of 75 patients were included and the GAMS and a dental examination were performed for validation.

**Results:**

The subjective assessment of patients and dental findings showed reduced oral health especially with recognized risk factors for the development of systemic comorbidities, such as dysphagia and malnutrition, whereas oral health problems were underestimated by patients. Bites, chewing problems and dry mouth showed sufficient correspondence between dental findings and subjective assessment.

**Conclusion:**

The GAMS could help facilitate the consideration of oral health problems in geriatric patients in a GP setting and promote cooperation with dentists in the sense of European recommendations for action.

Die Bedeutung der Mundgesundheit von Senioren nimmt in der Wahrnehmung von älteren Patienten, Pflegekräften, Hausärzten und Geriatern häufig eine untergeordnete Stellung ein, insbesondere wenn zunehmender Pflegebedarf und Multimorbidität eintreten [1]. Orale Erkrankungen haben aber gerade in dieser Population eine hohe Prävalenz und werden in engem Zusammenhang mit anderen somatischen Erkrankungen gesehen, wodurch sie zur Multimorbidität beitragen [1–5]. Das frühzeitige Erkennen von Kau- und Schluckproblemen spielt bei älteren, meist multimorbiden Patienten mit ohnehin erhöhtem Risiko für Dysphagie und Mangelernährung eine wichtige Rolle [6–8]. Mundtrockenheit stellt bei geriatrischen, meist polymedizierten Patienten mit altersphysiologisch eingeschränkter Speichelproduktion eine wichtige Medikationsnebenwirkung dar, die im klinisch-geriatrischen Alltag trotz ihrer negativen Auswirkungen auf Kau- und Schluckfunktion nicht routinemäßig im Rahmen geriatrischer Assessments erfasst wird [9]. Halitosis gilt seit Jahren als Epiphänomen für eine Reihe mundgesundheitsbezogener Probleme, insbesondere bei geriatrischen Patienten [10]. Die Multikausalität oraler Erkrankungen einerseits und die multidimensionalen Folgen andererseits, die von eingeschränkter Nahrungsaufnahme mit sekundärer Malnutrition über enorale Schmerzen und Kaubeschwerden bis hin zu Aspirationspneumonien mit erhöhtem Mortalitätsrisiko reichen, haben die Diskussion angeregt, eingeschränkte Mundgesundheit im Alter als eigenständiges geriatrisches Syndrom zu betrachten [11]. Dabei gibt es eine überzeugende Studienlage darüber, dass durch eine angemessene Mundpflege bis ins hohe Alter die Gesundheit sowohl von Mund als auch Gesamtorganismus positiv beeinflusst werden kann [12]. Es hat sich gezeigt, dass v. a. ambulant betreute Senioren mit zunehmendem Pflegebedarf den Anschluss an die zahnmedizinische Versorgung kontinuierlich verlieren [13]. Folge dieser Entwicklung ist eine eher problem- als präventionsorientierte Inanspruchnahme zahnärztlicher Dienste. Die weit verbreitete Empfehlung, alle 6 Monate einen Zahnarzt aufzusuchen, scheint für diese vulnerable Patientenpopulation kaum umsetzbar, da einerseits Mobilitätseinschränkungen ein Hindernis darstellen, andererseits aber auch die Möglichkeiten einer flächendeckend aufsuchenden mobilen zahnmedizinischen Versorgung zum jetzigen Zeitpunkt noch nicht ausreichend gegeben sind. Im Gegensatz zum Zahnarzt werden Hausärzte auch von hochaltrigen Patienten regelmäßig konsultiert und könnten daher als Schlüsselfaktor auch für auftretende zahnmedizinische Gesundheitsprobleme angesehen werden. Es gibt bisher im deutschsprachigen Raum weder geeignete validierte Erfassungsbogen von Mundgesundheit bei älteren Patienten im hausärztlichen Setting, noch gibt es einheitliche Empfehlungen zur Vorgehensweise, wie Mundgesundheit bei älteren Patienten von hausärztlicher Seite miterfasst werden könnte. In der Literatur ist das bekannteste Instrument zur Beurteilung der Mundgesundheit die Brief Oral Health Status Examination (BOHSE), die hauptsächlich für Pflegekräfte in Pflegeheimen entwickelt, validiert und angewendet wurde und sowohl Befragung als auch eine kurze orale Untersuchung beinhaltet [14]. Ebenso sind Oral Health Assessment Tool (OHAT) [15] und Mini Dental Assessment [16] Instrumente, die entweder nach einer zusätzlichen Person oder einer Form der objektiven Untersuchung verlangen. Die Erfahrung zeigt, dass die Untersuchung des Mundes für Nichtzahnärzte ein großes Hindernis darstellt. Sicherlich am erfolgversprechendsten hinsichtlich einer Verbesserung dieser Situation wären Modelle, die neben solchen Erhebungsinstrumenten auch relevante Aspekte wie die Schaffung von Strukturen verbesserter integrierter Versorgung oder entsprechende Anpassung von Ausbildungsinhalten zum Ziel haben.

Die Arbeitsgruppe um Gil-Montoya konnte in ihrem nichtsystematischen Übersichtsartikel nicht nur die enge Verknüpfung der häufigsten Mundgesundheitsprobleme mit geriatrischen Syndromen detailliert aufzeigen, sondern auch, daraus resultierend, den dringenden Bedarf nach einem ganzheitlichen Diagnostikansatz („comprehensive geriatric assessment“, CGA) unterstreichen, der neben biopsychosozialen und funktionellen Aspekten auch den oralen Gesundheitsstatus integriert [13]. Dieser Bedarf wird umso dringender hinsichtlich der wachsenden Datenlage über Präventionseffekte von effektiver Mundhygiene: So konnte durch Anwendung chlorhexidinhaltiger Mundpflegeprodukte das Risiko für Pneumonien bei Langzeitbeatmungen und krankenhausassoziierte Pneumonien („hospital-acquired pneumonias“) reduziert werden [17].

Während sich die theoretischen Kenntnisse über die enge Verknüpfung zwischen Mundgesundheit und systemischen Gesundheitsproblemen sowie geriatrischen Syndromen bei den meisten Akteuren im Gesundheitswesen erfreulicherweise immer weiterverbreiten und zu einer zunehmenden Formulierung von Handlungsempfehlungen führen, hinkt die praktische Umsetzung von Diagnostik und Therapie dagegen hinterher [18]. Hier werden Stimmen laut, die mehr interdisziplinäre Aus- und Weiterbildungskonzepte fordern [19, 20]. Bis diese Maßnahmen jedoch zum Tragen kommen, dauert es einige Zeit. Gegenwärtig werden die meisten ambulanten geriatrischen Patienten in erster Linie von ihren Hausärzten betreut, welche häufig aus den Fachbereichen Allgemeinmedizin oder innere Medizin kommen. Um den breiten Diagnostikbedürfnissen älterer Patienten begegnen zu können, hat die Deutsche Gesellschaft für Allgemeinmedizin und Familienmedizin 2018 die hausärztliche Leitlinie Geriatrisches Assessment in der Hausarztpraxis erarbeitet (https://www.awmf.org/uploads/tx_szleitlinien/053015l_S1_Geriatrisches_Assessment_in_der_Hausarztpraxis_2018-05-verlaengert.pdf.). Diese beinhaltet ein „Manageable Basis Assessment (MAGIC)“ für Hausärzte, basierend auf 9 obligat und 4 fakultativ abzufragenden Funktions- und Beschwerdebereichen. In dem fakultativen Beschwerdebereich „ungewollter Gewichtsverlust“ werden mundgesundheitsbezogene Aspekte dahingehend berücksichtigt, dass Hausärzte an einen schadhaften Zahnstatus denken sollten und eine zahnärztliche Untersuchung in Erwägung ziehen sollten. Ebenso wird auf eine medikamentöse Ursache für Mundtrockenheit verwiesen und eine Liste häufig verordneter Mundtrockenheit verursachender Medikamente aufgeführt (https://www.awmf.org/uploads/tx_szleitlinien/053015l_S1_Geriatrisches_Assessment_in_der_Hausarztpraxis_2018-05-verlaengert.pdf). Empfehlungen über die Klärung der Frage nach Dringlichkeit einer zahnärztlichen Untersuchung finden sich in der Leitlinie jedoch ebenso wenig wie ein strukturiertes Vorgehen zur Basisdiagnostik von Mundgesundheitsproblemen. In diesem Gesamtkontext verfolgt unsere Arbeitsgruppe in dieser Untersuchung das Ziel, die Mundgesundheit anhand eines rein subjektiven Fragebogens zu beurteilen, der den Patienten ausgehändigt werden kann und nur durch eine hausärztlich-geriatrische Einschätzung hinsichtlich der Dringlichkeit eines Zahnarztbesuches abgerundet wird, um die Hürde für Nichtzahnärzte und den zeitlichen Aufwand möglichst gering zu halten. Ein solcher Fragebogen könnte sowohl Patienten als auch Hausärzte für die Mundgesundheit sensibilisieren und zudem als Rechtfertigung für eine bedarfsorientierte zukünftig mögliche Überweisung zum Zahnarzt dienen.

Ziele dieser Studie waren daher die Entwicklung, Erprobung und Validierung eines subjektiven Mundgesundheitsfragebogens durch Patienten in einem ambulanten Praxissetting in Kombination mit einer Dringlichkeitseinschätzung hinsichtlich Vorstellung des Patienten beim Zahnarzt (Geriatrisches Ambulantes Mundgesundheits-Screening, GAMS).

## Studiendesign und Untersuchungsmethoden

### Entwicklung von Fragebogen/Einzelitems.

In dieser Studie soll ein Erhebungsbogen für Allgemeinmediziner entwickelt werden, der die häufigsten zahnmedizinischen Probleme mit notwendigem zahnmedizinischen Handlungsbedarf bei Senioren abbildet und ohne zahnmedizinische Fachkenntnisse erhoben werden kann sowie keine klinische Munduntersuchung erfordert. Der zeitliche Aufwand sollte möglichst gering gehalten werden, um den Bogen in die tägliche Praxisroutine einbauen zu können. Dieser Fragebogen soll keine zahnmedizinische Untersuchung und Therapie ersetzen, sondern dem Hausarzt Hinweis darauf geben, ob der Patient eher zeitnah oder aber im geplanten regulären Intervall einem Zahnarzt vorgestellt werden sollte. Bei der Entwicklung des Fragebogens wird besonderes Augenmerk auf zahnmedizinische Mundgesundheitsprobleme gelegt, die in dieser Patientengruppe häufig vorkommen und zur Vermeidung von Folgestörungen einer zahnmedizinischen Therapie dienen. Hierzu wurden nach entsprechender Literaturrecherche und Definition des aktuellen Standes der Literatur [10] die folgenden Teilbereiche festgelegt (Tab. 1). Für jedes der beschriebenen zahnmedizinischen Gesundheitsprobleme wurde eine Frage entwickelt, die das beschriebene zahnmedizinische Gesundheitsproblem darstellen soll. Zudem wurde eine Gesamtfrage zur Mundgesundheit beigefügt, um überprüfen zu können, ob mit einer Frage bereits derselbe Nutzen erzielt werden könnte wie durch die individuellen themenorientierten Fragen.

**Table 1:** 

	Mundgesundheitsproblem	Zahnmedizinischer Untersuchungsparameter
*Haben Sie Gesundheitsprobleme im Mundbereich?*	*Subjektive Gesamtmundgesundheit*	–
1. Leiden Sie an Missempfindungen wie Brennen oder pelzigem Gefühl im Mund?	Mundschleimhautprobleme	Druckstellen Einbisse Candidiasis Rhagaden
2. Haben Sie Schmerzen oder andere Beschwerden im Bereich von Mund und Zähnen?	Kariöse Läsionen, Kronenfrakturen, zerstörte Zähne	Kariös Zerstört Druckstellen Einbisse Candidiasis Rhagaden
3. Haben Sie Zahnfleischbluten beim Putzen, beim Essen oder auch spontan?	Gingivitis, Parodontitis, mangelhafte Mundhygiene	Approximaler Plaque-Index Sulcus-Blutungsindex Gesamt-PSI Höchster PSI-Wert
4. Haben Sie Schwierigkeiten beim Zerkauen und beim Schlucken von Möhren und Äpfeln?	Reduzierte Kaufunktion	Reduzierte Zahnzahl Kariös Zerstört Ersetzt Xerostomie Unstimulierte Speichelfließrate Stimulierte Speichelfließrate
5. Ist Ihnen selbst ein unangenehmer Geruch aus dem Mund aufgefallen, oder hat Sie jemand darauf angesprochen?	Halitosis	Organoleptischer Index (0–3)
Haben Sie öfter das Gefühl, dass Ihr Mund trocken ist?	Mundtrockenheit	Xerostomie Unstimulierte Speichelfließrate Stimulierte Speichelfließrate
6. Haben Sie Voll- oder Teilprothesen?	Vorhandene Prothetik mit regelmäßiger Notwendigkeit einer zahnärztlichen Kontrolle	Prävalenz prothetischer Versorgung

Der Fragebogen wurde hinsichtlich der Formulierungen in Anlehnung an die etablierten englischsprachigen BOHSE-Fragebogen [14] entwickelt, der bisher allerdings nicht auf Deutsch existiert. Zielgruppe dieser Untersuchung waren dabei nicht die geriatrischen Patienten mit akuten Zahnschmerzen, welche sich problemorientiert in die zahnärztliche Behandlung begeben. Zielgruppe waren vielmehr diejenigen geriatrischen Patienten, bei welchen das Vorliegen eines elektiven zahnmedizinischen Handlungsbedarfes überprüft werden soll. Zur möglichst einfachen und schnellen Durchführbarkeit im nichtzahnärztlichen Setting reduzierten wir den Fragenumfang auf 6 Kernfragen wurde der Fragenumfang auf 6 Kernfragen reduziert. Die Anwendbarkeit und Verständlichkeit wurden in einer Vorlaufphase von jeweils 10 Alterszahnmedizinern, 10 Hausärzten und 10 Geriatern überprüft. Dabei ergaben sich folgende Kritikpunkte:

*positiv:* Fragebogen ist verständlich, gut einsetzbar; der zeitliche Umfang angemessen; er ist in das geriatrische Assessment integrierbar;

*negativ*: Wie kann eine Vorstellung beim Zahnarzt sichergestellt werden? Was passiert mit mobilitätseingeschränkten Patienten und zahnärztlichem Handlungsbedarf?

Die Einteilung der Dringlichkeitsbewertung erfolgte empirisch auf Basis der klinischen Empfehlung der befragten Alterszahnmediziner. Hierbei wurde davon ausgegangen, dass akute Schmerzsymptomatiken im Mund von Patienten als Grund angesehen werden, zügig einen Zahnarzt aufzusuchen, und im Rahmen der Abfrage innerhalb des Assessments eher chronische Schmerzzustände adressiert werden sollen.

## Ein- und Ausschlusskriterien

In diese Studie wurden nur Personen, die ambulant hausärztlich betreut werden, eingeschlossen, aufgrund des zahnärztlichen Fokus wurde „alt sein“ entsprechend den Richtlinien der Deutschen Mundgesundheitsstudie V als >75 Jahre definiert [13]. Zusätzliche Einschlusskriterien waren die Bereitschaft, sich innerhalb einer Woche zahnärztlich untersuchen zu lassen, sowie der Ausschluss von Demenz entsprechend der vorhandenen Facharztdiagnose.

## Hausärztliches Vorgehen

Den 75 eingeschlossenen, ambulant betreuten Patienten wurde der Fragebogen zur Beantwortung im Wartezimmer gegeben. Im Einzelfall wurden die Patienten durch medizinisches Assistenzpersonal beim Ausfüllen unterstützt, wobei die Vorstellung in der hausärztlichen Praxis im Rahmen eines medizinisch indizierten Besuches erfolgte. Alter, BMI, Komorbiditäten und ggf. aktualisierter Medikationsplan konnten aus der Krankenakte entnommen werden. Die Diagnose eines Diabetes mellitus wurde im Vorfeld gestellt, wenn in der laborchemischen Untersuchung der Nüchternwert für Glucose mindestens einmalig einen Wert über 126 mg/dl gezeigt hatte oder der HbA_1c_-Wert ≥6,5 % war. Hierbei ist kritisch anzumerken, dass es im Falle einer späteren Unterschreitung der Werte nicht zu einer Rückcodierung der Variable Diabetes kam, sodass möglicherweise überpositive Prävalenzen berichtet wurden. Die Durchsicht des bereits vorausgefüllten Bogens wurde im Behandlungszimmer durch den Hausarzt durchgeführt, und anhand der gegebenen Patientenantworten wurde die Dringlichkeit eines Zahnarztbesuches folgendermaßen bewertet:

1. Der Patient kreuzt nur „nein“ angekreuzt und ein Zahnarztbesuch innerhalb der letzten 6 Monate: kein Grund zur Überweisung zum Zahnarzt.
2. Der Patient kreuzt ein- bis 2‑mal „ja“ angekreuzt und ein Zahnarztbesuch liegt länger als 3 Monate zurück: es besteht Grund zur Überweisung zum Zahnarzt in 2 Monaten.
3. Der Patient kreuzt 3‑ bis 4‑mal „ja“ angekreuzt: der Patient sollte in den nächsten 2 Wochen einen Termin beim Zahnarzt vereinbaren.
4. Der Patient kreuzt mehr als 4‑mal „ja“ angekreuzt oder akute Schmerzen: sofortiger Termin beim Zahnarzt empfohlen.

Da weder eine formgerechte noch eine formlose Überweisung vom Hausarzt zum Zahnarzt aktuell berufsrechtlich zulässig ist, wurde dem Patienten ausschließlich eine mündliche Empfehlung, sich zahnärztlich vorzustellen, ausgesprochen.

## Zahnmedizinische Untersuchung

Die Validierung des Instrumentes erfolgte in einem weiteren Schritt durch eine zahnärztliche Untersuchung und Überprüfung der hausärztlichen Dringlichkeitseinschätzung. Die subjektiven Einzelitems sowie die zusammenfassende Einzelfrage sollten hierbei verglichen werden, um die mögliche Übereinstimmungen zu dokumentieren. Die zahnmedizinischen Untersuchungsbefunde als objektive Parameter wurden mit der subjektiven Einschätzung der Patienten (Tab. 1) verglichen. Eine Darstellung der zahnmedizinischen Parameter erfolgt im Zusatzmaterial online: „Zahnmedizinische Untersuchungsbefunde“.

## Statistische Auswertung

Nach der Datenaufbereitung erfolgte eine deskriptive Analyse zur Beschreibung des Kollektivs. Dabei wurden stetige normal verteilte Merkmale mittels Mittelwert mit Standardabweichung und nichtnormal verteilte als Median mit Interquartilsabstand angegeben. Kategoriale Merkmale wurden als absolute und relative Häufigkeiten in Prozent berichtet. Zum Vergleich der subjektiven Befunde mittels Fragebogen und objektiven zahnärztlichen Befunde wurden die Merkmale mittels Kreuztabellen untersucht. Weiterhin wurden kategoriale Merkmale mittels Übereinstimmungsgüte, die sog. Interrater-Reliabilität, auf Zusammenhänge untersucht und mit dem Cohens κ als statistisches Maß berichtet. Kappa kann Werte zwischen −1 ≤ κ ≤ 1 annehmen. Dabei gilt ein κ < 0 nach Landis und Koch [21] als schlechte Übereinstimmung („poor“), für 0 ≤ κ ≤ 0,2 spricht man von geringer Übereinstimmung („slight“), zwischen 0,21–0,40 von mäßiger Übereinstimmung („fair“), für 0,41–0,60 von moderater Übereinstimmung („moderate“), zwischen 0,61 und 0,80 von substanzieller Übereinstimmung („substantial“) sowie für 0,81–1,00 von (fast) perfekter Übereinstimmung („almost perfect“). Als Signifikanzniveau wurde ein α von 0,05 gewählt. Ein *p*-Wert kleiner 5 % wurde als statistisch signifikant angesehen. Alle Analysen wurden mit der Statistiksoftware SPSS 25 von IBM durchgeführt.

## Ethikvotum

Die Studie wurde unter der Nummer 18-324 durch die Ethikkommission der Universität Köln positiv bewertet. Eine Studienregistrierung beim DRKS (www.drks.de) erfolgte unter der Nummer DRKS00015630. Die Studie wurde entsprechend der Deklaration von Helsinki/Good clinical practice durchgeführt.

## Ergebnisse

### Klinische Charakteristika.

Die Studienteilnehmer waren zu 60 % weiblich, 40 % männlich. Die Teilnehmer zeigten eine hohe Prävalenz der Einnahme von Antiphlogistika (82 %) und Diabetes mellitus Typ 2 (82 %). Mittleres Alter war 80 (SD 4), im Mittel wurden 10 (SD 5) Komorbiditäten angegeben und 6 (SD 4) Medikamente täglich eingenommen (Tab. 2). Der letzte Zahnarztbesuch lag im Mittel über ein Jahr zurück.

**Table 2:** 

	Gesamt (*n* = 75)
Geschlecht (weiblich)	45 (60)
Alter in Jahren	80 ± 4
Größe in cm	168 ± 7
Gewicht in kg	76 ± 13
BMI	27 ± 4
Antiphlogistikaeinnahme	31 (82)
Rheumatoide Arthritis	4 (10)
Diabetes Typ 2	29 (82)
Hauszahnarzt vorhanden	66 (88)
Polypharmazie (>5)	39 (52)
Anzahl der Diagnosen, Komorbiditäten	9 (7; 14)
Anzahl der Medikamente	6 ± 4

Kategoriale Merkmale werden als abs. Häufigkeiten mit Prozent *n* (%), normal verteilte stetige Merkmale als Mittelwert ± Standardabweichung, nichtnormal verteilte Merkmale als Median mit Interquartilsabstand (IQR_0,25_; IQR_0,75_) angegeben

### Geriatrisches ambulantes Mundgesundheits-Screening.

Hinsichtlich der eigenen subjektiven Einschätzung ihrer Mundgesundheit zeigten sich insbesondere bei den Teilbereichen „Probleme beim Kauen oder Schlucken“ (19 %), „Mundgeruch“ (12 %), „Mundtrockenheit“ (40 %) sowie dem Teilbereich „Vorhandensein von Prothesen“ (73 %) auffällige Befunde. Die Beantwortung der Frage zu Problemen hinsichtlich der Gesamtmundgesundheit wurde von 15 % der Studienteilnehmer bejaht (Tab. 3). Wenn die Einzelitems anhand von Kreuztabellen in Relation zu der Gesamtfrage „Probleme im Mundbereich“ gestellt wurden, bestand bei folgenden Items eine gute Übereinstimmung (<5 %-Angabe eines Problems beim Einzelitem, aber keine Bejahung des Gesamtitems „Probleme im Mundbereich“), durch die Patienten: „Missempfindung/Brennen“ (3 %), „Schmerzen im Mund“ (3 %), „Zahnfleischbluten“ (4,7 %).

**Table 3:** 

	Gesamt (*n* = 75)
Prothesendruckstellen	14 (19)
Rhagaden	12 (16)
Visuelle *Candida*-Besiedlung	2 (3)
Hyposalivation, unstimuliert (<0,2 ml/min)	14 (19)
Hyposalivation, stimuliert (<0,7 ml/min)	14 (19)
Halitosis (Organoleptischer Score >0) [22]	57 (76)
API	50 (24; 100)
SBI	11 (0; 37)
DHI	2 (0; 8)
Zahnzahl	14 ± 10
Ersetzte Zähne	9 (0; 25)
PSI_max_	2,7 ± 0,9
PSI_mean_	2,3 ± 0,7

*API* approximaler Plaque-Index, *SBI* Sulcus Bleeding Index, *DHI* Denture Hygiene Index

Kategoriale Merkmale werden als abs. Häufigkeiten mit Prozent *n* (%), normal verteilte stetige Merkmale als Mittelwert ± Standardabweichung, nichtnormal verteilte Merkmale als Median mit Interquartilsabstand (IQR_0,25_; IQR_0,75_) angegeben

Dahingegen bestand eine geringere Übereinstimmung bei der subjektiven Bewertung der „Probleme beim Kauen und Schlucken“ (17 %), „Mundgeruch“ (8 %), „Mundtrockenheit“ (38 %). Das bedeutet, dass bei Vorliegen dieser Probleme diese nicht als Problem im Mundbereich abgebildet wurden. Zudem wurde eine Variable erstellt, bei der die Einzelitems als Mundprobleme zusammengefasst wurden (ja, wenn mindestens eines der Probleme, und nein, wenn keines der Probleme angegeben wurde). 53 Patienten (83 %) haben die globale Frage mit nein beantwortet, obwohl sie mindestens eines der Einzelprobleme mit ja beantwortet haben.

### Zahnmedizinischer Befund.

Im zahnärztlichen Untersuchungsbefund waren folgende Befunde auffällig (Tab. 3): Druckstellen unter Prothesen, Rhagaden, Speichelmangel. Die Studienteilnehmer zeigten parodontalen Behandlungsbedarf, eine mittlere Zahnzahl von 14 (SD 10) und eine mangelnde Prothesenhygiene.

### Dringlichkeitseinschätzung des Zahnarztbesuches.

Während der umgehende Zahnarztbesuch vom Hausarzt bei 1 % [1] der Studienteilnehmer angenommen wurde, war dieser bei 3 % [2] bei der Untersuchung durch den Zahnarzt indiziert. Die Vorstellung in den nächsten 2 Wochen wurde durch den Hausarzt in 9 % [7] der Fälle vs. 28 % [17] der Fälle durch den Zahnarzt bewertet und die Vorstellung beim Zahnarzt innerhalb der nächsten 2 Monate bei 63 % [*N* = 47] bewertet durch den Hausarzt vs. 56 % [*N* = 42] durch den Zahnarzt. Bezüglich der notwendigen sofortigen Vorstellung beim Zahnarzt aus Hausarztsicht wurde dies bei 3 % durch den Zahnarzt als nicht so dringlich angesehen; bei der Vorstellung innerhalb der nächsten 2 Wochen erfolgte in 24 % der Fälle eine Unterschätzung durch den Hausarzt und bei der Vorstellung innerhalb der nächsten 2 Monate eine 43 %ige differente Einschätzung. Zusammenfassend wurde die Notwendigkeit eines Zahnarztbesuches durch den Zahnarzt als dringlicher eingeschätzt als durch den Hausarzt.

### Übereinstimmung der subjektiv erhobenen Einzelitems des GAMS mit den objektiven zahnmedizinischen Befunden, Interrater-Reliabilität.

Hinsichtlich der Cohens-κ-Werte zeigten die Einbisse ausreichende Übereinstimmung mit dem subjektiven Schmerzempfinden. Ebenso ausreichende Übereinstimmung bestand zwischen der „Schwierigkeit, Äpfel und Möhren zu kauen“ und dem „Vorhandensein ersetzter Zähne“ sowie „Xerostomie“. Zudem zeigt die im Fragebogen angegebene Mundtrockenheit ausreichende Übereinstimmung mit der Einschätzung des Zahnarztes (Tab. 4).

**Table 4:** 

Subjektive Einschätzung *n* (%)	Objektiver zahnmedizinischer Befund	Cohens κ
Leiden Sie an Missempfindungen wie Brennen oder pelzigem Gefühl im Mund? 3 (4)	Druckstellen	−0,071
	Einbisse	−0,053
	Candidiasis	−0,033
	Rhagaden	0,074
Haben Sie Schmerzen oder andere Beschwerden im Bereich von Mund und Zähnen? 7 (9)	Druckstellen	−0,033
	Einbisse	**0,277**
	Candidiasis	−0,043
	Rhagaden	0,105
	Kariöse Zähne	0,075
	Zerstörte Zähne	−0,043
Haben Sie Zahnfleischbluten beim Putzen, beim Essen oder auch spontan? 6 (8)	Approximaler Plaque-Index	
	Sulcus-Blutungsindex	
	Gesamt-PSI	
	Mittlerer höchster PSI-Wert	0,044 0,033
Haben Sie Schwierigkeiten beim Zerkauen und beim Schlucken von Möhren und Äpfeln? 14 (19)	Zahnzahl	–
	Ersetzte Zähne	**0,217**
	Kariöse Zähne	0,034
	Unstimulierte Speichelfließrate	0,122
	Stimulierte Speichelfließrate	0,122
	Xerostomie	**0,208**
	Totalprothese	–
Ist Ihnen selbst ein unangenehmer Geruch aus dem Mund aufgefallen, oder hat Sie jemand darauf angesprochen? 9 (12)	Organoleptischer Index (0–3)	0,083
Haben Sie öfter das Gefühl, dass Ihr Mund trocken ist? 30 (40)	Xerostomie	**0,318**
	Stimulierte Speichelfließrate	0,085
	Unstimulierte Speichelfließrate	0,146

*Fett* gedruckte Werte repräsentieren statistisch signifikante Korrelationen (p < 0,05).

## Diskussion

In diesem Projekt wurden die Entwicklung und Validierung des GAMS zur Erfassung der Mundgesundheit von ambulant betreuten Senioren durch Hausärzte und Geriater beschrieben. Hierbei wurden in der Gruppe der Senioren hochprävalente zahnmedizinische Teilbereiche definiert und diesen Teilbereichen einfache für Patienten im täglichen Praxisablauf in einer Hausarztpraxis beantwortbare binäre Fragen zugeordnet. Zudem wurde eine Frage zur Gesamtmundgesundheit entwickelt und hinsichtlich ihrer Aussagekraft überprüft. Es erfolgte zusätzlich die zahnmedizinische Evaluation der Mundgesundheit und somit Validierung der subjektiven genannten Antworten.

Die sowohl bei der subjektiven Einschätzung der Patienten als auch bei der zahnmedizinischen Untersuchung diagnostizierten Mundgesundheitseinschränkungen wie „Probleme beim Kauen oder beim Schlucken“, „Mundgeruch“ und „Mundtrockenheit“ weisen auf die im geriatrischen Kollektiv besonders häufig auftretenden Mundgesundheitsprobleme hin, welche inzwischen anerkannte Risikofaktoren für die Entwicklung systemischer Komorbiditäten und Folgestörungen sind, wie Dysphagie, Mangelernährung oder Pneumonie [23–25]. Der von uns entwickelte GAMS könnte hier ansetzen und dazu beitragen, die Erwägung und Berücksichtigung von Mundgesundheitsproblemen bei ambulanten geriatrischen Patienten im hausärztlichen Setting zu erleichtern und die Zusammenarbeit mit Zahnmedizinern im Sinne europäischer Handlungsempfehlungen zu fördern. Zur optimalen Betreuung multimorbider geriatrischer Patienten ist ein ganzheitlicher Diagnostik- und Therapieansatz auf der Basis des multidimensionalen geriatrischen Assessments notwendig. Die interdisziplinäre Zusammenarbeit mit Fachgruppen wie Ergotherapie, Physiotherapie, Logopädie und Neuropsychologie wird heute sogar durch die Abrechnungsmodalitäten der geriatrischen Komplexbehandlung berücksichtigt. Inzwischen gibt es auch zertifizierte Kooperationszentren zwischen Geriatern und Alterstraumatologen. Kooperationen zwischen Hausärzten und Fachärzten werden im Sinne einer guten Patientenversorgung im Vertragsarztänderungsgesetz [26], das seit 2007 in Kraft ist, sogar ausdrücklich gefördert, sei es in Form von Berufsausübungsgemeinschaften oder innerhalb medizinischer Versorgungszentren (MVZ). Bedauerlicherweise sind jedoch Kooperationen zwischen Vertragshausärzten und Vertragszahnärzten davon ausgeschlossen; eine Praxisgemeinschaft oder eine Berufsausübungsgemeinschaft ist nicht möglich. Auch die noch immer weitgehend getrennte Ausbildung von Human- und Zahnmedizinstudenten verhindert eine gerade im geriatrischen Setting dringend notwendige spätere Zusammenarbeit aufgrund unzureichender Fachkenntnisse und eines dadurch oft eingeschränkten Stellenwerts in der Beurteilung des jeweils anderen Fachbereiches. Aufgrund der hohen Relevanz mundgesundheitsbezogener Defizite mit Einfluss auf die Allgemeingesundheit geriatrischer Patienten sind feste interdisziplinäre Versorgungsstrukturen nicht nur wünschenswert, sondern für eine optimale Betreuung auch essenziell. Bis die entsprechenden kooperationserleichternden Strukturen geschaffen sind, können Screeningbogen wie der GAMS zu einer Sensibilisierung für mundgesundheitsbezogene Probleme unter Haus- und Fachärzten beitragen und frühzeitig auf Defizite aufmerksam machen. Die Kontaktaufnahme zum Zahnarzt muss vorerst aufgrund fehlender Strukturvorgaben über persönliche Netzwerkbildung laufen, ergänzt durch datenschutzwahrende erweiterte Kommunikation (z. B. durch das Versenden von Befunden).

Ein mundgesundheitsbezogenes Screening im hausärztlichen Setting kann zudem zu einer Verbesserung der Patientenzufriedenheit beitragen. Eine Freiburger Literaturanalyse ergab 2016, dass interprofessionelle Zusammenarbeit nicht nur zu einer größeren Zufriedenheit zwischen den Professionen beitrug, sondern auch zu einer verbesserten Patientenzufriedenheit führte [27]. So kann eine hausärztliche Frage zur prothetischen Versorgung bereits auf ein Versorgungsdefizit und einen Versorgungsbedarf unter älteren Patienten hinweisen, wie die Ergebnisse unserer Untersuchung ergaben:

Unsere subjektive Befragung hinsichtlich vorhandener Prothesen ergab eine hohe Prävalenz. Basierend auf unseren klinischen Erfahrungen ist hierbei problematisch, dass viele Patienten das Vorhandensein von Prothesen nicht mit einer notwendigen regelmäßigen Kontrolle beim Zahnarzt assoziieren, während der Hausarzt durchaus weiteraufgesucht wird. Eine regelmäßige Nachsorge hinsichtlich Funktionalität, Passform, hygienischer Situation ist notwendig, wird aber von den Patienten häufig vernachlässigt. Hier kann eine Abfrage allein der Prävalenz und der damit verbundenen Aufforderung, einen Zahnarzt regelmäßig aufzusuchen, helfen, mögliche Probleme frühzeitig zu identifizieren und zu beheben.

Unser auf interprofessionelle Zusammenarbeit ausgerichteter Screeningbogen GAMS würde das Spektrum der bereits existierenden interdisziplinären Assessments erweitern: So sind beispielweise Assessmentinstrumente für ein Diabetesscreening, die in der Zahnarztpraxis angewendet werden können, beschrieben worden (FINDRISK) [28]. Der GAMS mag ein entsprechender erster Schritt zu einem Assessmentinstrument zur Vorstellung bei Zahnarzt sein. Dafür müssten aber, wie bereits erwähnt, die aktuellen berufsrechtlichen Rahmenbedingungen angepasst werden, damit eine Überweisung vom Hausarzt zum Zahnarzt zulässig wird. Die durch uns etablierte Gesamtfrage nach Problemen im Mundbereich bildet die Häufigkeit der Beschwerden, erfragt durch Einzelitems, nicht ab, sodass eine differenziertere Abfrage notwendig erscheint. Wie in der Literatur beschrieben, werden einzelne der in den Einzelitems abgefragten Probleme von Patienten nicht als mundgesundheitsrelevante Probleme eingeschätzt, und diese werden häufig nicht berichtet. Daher schätzen wir die individuelle Abfrage als zielführender ein als eine Gesamtfrage, die die hohe Prävalenz von Mundgesundheitsproblemen nicht abbildet.

Hinsichtlich der Dringlichkeit wurde die Notwendigkeit einer zahnärztlichen Vorstellung leicht zeitkritischer eingeschätzt als durch den Hausarzt, in den meisten Fällen war die Einschätzung aber identisch. Natürlich wäre die korrekte Einordnung der Dringlichkeit auf hausärztlicher Seite wünschenswert, da dadurch eine Gefahr der Unterbehandlung vermieden werden könnte. Dennoch entsteht aufseiten der Hausärzte keine Verantwortlichkeit zu einer korrekten Einschätzung bzw. keine Verantwortlichkeit zu einer Überweisung. Der Bogen soll ausschließlich einer Unterstützung dienen und im hausärztlichen Bereich bei der Betreuung geriatrischer Patienten für Mundgesundheitsprobleme sensibilisieren. Es kann daher diskutiert werden, ob nicht auch eine reine Abfrage der Zeitspanne seit dem letzten Zahnarztbesuch des Patienten gereicht hätte, um eine Vorstellung zu rechtfertigen. Dennoch erscheint die hohe Relevanz von Mundgesundheitsproblemen mit Einfluss auf die Allgemeingesundheit im höherem Alter als Rechtfertigung dafür, diese auch differenziert abzufragen und in die hausärztliche Therapieplanung einzubauen.

Hinsichtlich der Interrater-Reliabilität zwischen Zahnarzt und der subjektiven Einschätzung kann ebenfalls nur bei den relevanten Mundgesundheitsproblemen subjektives Schmerzempfinden, Kauschwierigkeiten, ersetzte Zähnen sowie Mundtrockenheitsproblematik ausreichende Übereinstimmung gezeigt werden. Dies mag ebenfalls zum einen an der Unterschätzung der Mundgesundheitsprobleme der Patienten im Kontext einer reduzierten Allgemeingesundheit mit anderen relevanten und stärker subjektiv beeinträchtigenden Gesundheitsproblemen liegen. Zum anderen kann es aber auch durch die relativ kleinen Stichprobengrößen bedingt sein, denn bei höheren Prävalenzen (*n* > 30) wurden die κ‑Übereinstimmungswerte deutlicher.

Grundsätzlich müssen in der Diskussion zudem diejenigen Aspekte der Versorgung, die über die direkte Versorgung durch Zahn- und Hausärzte hinausgehen, sich aber dennoch in mangelnder Mundgesundheit ausdrücken, wie etwa ungedeckte Pflege- und Rehabilitationsbedarfe, berücksichtigt werden. Ganz grundsätzlich sind Senioren mit zunehmender Pflegebedürftigkeit weniger belastbar: Therapiefähigkeit, Mundhygienefähigkeit und Eigenverantwortung nehmen ab, und ein Drittel der Menschen mit Pflegebedarf kann sich nicht mehr eigenständig um die Mund- und Prothesenhygiene kümmern und benötigt Hilfe [13]. Multiple systemische Erkrankungen, Multimorbidität, Demenz, reduzierte Mobilität und häufige Polypharmazie verschlechtern und potenzieren die Situation zusätzlich und hindern sie selbst und das Pflegepersonal zunehmend, gute Mundhygienemaßnahmen durchzuführen [17, 18]. So stellt allein die Tatsache, dass jemand für Mundhygienemanöver externe Hilfe benötigt, ein erhöhtes Risiko für eine reduzierte Mundgesundheit dar. Die daher geforderte Unterstützung in späteren Lebensphasen bei der Mundpflege durch das Pflegepersonal soll die reduzierte Mundhygienefähigkeit der Patienten ersetzen. Ob dies bei den beschriebenen Risikofaktoren selbst bei perfekter Durchführung zum Erhalt guter Mundgesundheit ausreichen kann, bleibt fraglich. Zudem werden in der Literatur aufseiten der Pflegekräfte organisatorische Missstände angegeben: zu wenig Zeit, häufige Personalwechsel, aber auch wenig eigenes Gesundheitsbewusstsein im Hinblick auf die eigene Mundhöhle. Häufig wird mangelnde Ausbildung der Pflegekräfte in Bezug auf Mundhygieneprävention im Heim genannt, allerdings auch fehlende zahnärztliche Ansprechpartner sowie der Mangel an wirksamen Strategien, dem Unwillen zur Mitarbeit bei den Bewohnern zu begegnen [19, 20, 29, 30].

## Limitationen

Da diese Pilotuntersuchung zunächst als monozentrische Untersuchung in einer Praxis durch einen Hausarzt und durch eine Zahnärztin überprüft wurde, in nur einem schon langfristig kontinuierlich angebundenen Patientenkollektiv, ist die externe Validität der Daten begrenzt. Eine Validierung des Fragebogens mit multizentrischem Einsatz und höheren Patientenzahlen sollen der Inhalt einer Folgeuntersuchung werden. Ebenso stellt sich hinsichtlich der praktischen Umsetzung die Frage, ob vonseiten der Hausärzte ein solches Instrument praktikabel erscheint und in welcher Frequenz dieses angewandt werden müsste, um wirksam zu sein. Auch dies sollte Teil weiterführender Untersuchungen sein.

## Fazit für die Praxis

Der Einsatz eines Assessmentinstruments zur subjektiven Mundgesundheit zum Einsatz in der hausärztlichen Routine, das relevante und hochprävalente Mundgesundheitsparameter bei Senioren und geriatrischen Patienten abbildet, kann Hausärzten, Geriatern und Patienten helfen, Mundgesundheitsprobleme zu identifizieren. Da der Hausarzt auch für hochaltrige Patienten gewöhnlich der erste Ansprechpartner bleibt, diese Patienten aber aus der zahnärztlichen Versorgung immer stärker herausfallen, kann durch ein solches Assessmentinstrument zudem die Patientensensibilität für Mundgesundheit erhöht werden, bis hin zu regelmäßigen kontroll- und präventionsorientierten Terminen. Entsprechend den europäischen Forderungen sollte bei der Behandlung geriatrischer Patienten angestrebt werden, die strikte Trennung der Fächer Zahnmedizin und Medizin zu überwinden, um im Sinne der Patienten eine interdisziplinäre Betreuung zumindest von sich beidseitig beeinflussenden Gesundheitsproblemen zu ermöglichen.

## Caption Electronic Supplementary Material


