# Supplementary material for: Oral health assessment of seniors under outpatient care by family doctors: Development and validation of the geriatric outpatient oral health screening
Source: Z Gerontol Geriatr. 2020 Apr 29;54(3):255–63. [Article in German] doi: 10.1007/s00391-020-01730-5 (PMC8096727; doi:10.1007/s00391-020-01730-5)
Supplement: Supplementary file 1 [file 391_2020_1730_MOESM1_ESM.docx]

**Supplementary materials 1**. Zahnmedizinische Untersuchungsbefunde.

Neben einer umfassenden visuellen intraoralen Schleimhautuntersuchung wurde das individuelle Kariesrisiko anhand des sogenannten dmft-Indexes ermittelt: d – decayed (zerstörte), m – missing (fehlende), f – filled (gefüllte), t – teeth (Zähne) bzw. S = surfaces (Flächen) im bleibenden Gebiss (DMF-T/ DMF-S).

Die subjektiv empfundene Mundtrockenheit wurde mittels einer binären ja/nein-Frage abgefragt. Zudem wurden die unstimulierten und stimulierten Speichelfließraten wie in der Literatur beschrieben gemessen. Hyposalivation wurde definiert ab einer unstimulierten Speichelfließrate <0,2ml/min und einer stimulierten Speichelfließrate <0,7ml/min.

**API.** Erhoben wurde zudem der Approximale Plaque-Index (API) als Messzahl zur Kontrolle der [Mundhygiene](https://www.zahnarzt-zahnbehandlung.com/index.php/mund-pflege/mund-hygiene). Vorhandene Plaque wird durch Färbung sichtbar gemacht und nach visuellen Gesichtspunkten beurteilt. Der API ist somit die Messung der Plaqueansammlung pro Zahnzwischenraum nach Anfärben. Die Summe verschmutzter Flächen in Relation zur Summe der untersuchten Flächen ergibt den API. Hinsichtlich der Beurteilung werden API-Werte <35% als ausreichend angesehen, wohingegen Werte >35% verbesserungswürdig sind (Lange 1975).

**SBI.** Der modifizierte Sulcus-Blutungs-Index (nach Mühlemann und Son 1975, Lange 1990) (SBI) beurteilt das Vorkommen von Blutungen in den Zahnzwischenräumen ohne weitere Graduierung als Entzündungsindikator. Beim SBI >20% gilt das Parodont als behandlungsbedürftig. Ziel der Mundhygiene ist ein SBI von unter 10 %.

**PSI.** Das gesamte Gebiss wird Zahn für Zahn (unter Einbeziehung von Implantaten) untersucht. Zur Erhebung ist das Gebiss in Sextanten eingeteilt. Die Sonde wird distal in den Sulkus des letzten Zahnes eingeführt und um den gesamten Zahn herumbewegt. Die möglichen Befunde sind in den Codes 0 bis 4 zusammengefasst. Pro Sextant wird nur der höchste Wert notiert. Ab **Code 1** liegt eine Gingivitis, bei Codezahlen ab 3 eine Parodontitis vor und weitergehende diagnostische und therapeutische Maßnahmen im betroffenen Sextanten werden erforderlich. Sind zwei und mehr Sextanten mit Code 3 bewertet, empfehlen sich weitergehende Maßnahmen für das gesamte Gebiss.

**DHI.** Dieser Index stellt eine einfache Befunddokumentation der Prothesenpflege dar und soll neben der Dokumentation auch der Verlaufskontrolle dienen (Range 1-10). Ab einem DHI Wert > 2 ist die Prothesenpflege verbesserungswürdig.

**Organoleptischer Score.** Es erfolgt in der täglichen Praxis die Unterteilung in 4 Schweregrade. Dabei hält der Untersucher seine Nase in einem definierten Abstand zum Patientenmund. Der Patient wird gebeten den Vokal ”A“ zu sprechen. Kann der Untersucher den Mundgeruch im Abstand von 1m wahrnehmen, liegt der Schweregrad 3, bei einem Abstand von 30 cm liegt ein Schweregrad 2, und bei einem Abstand von 10 cm liegt der Schweregrad 1 vor.
